# PET-guided treatment in advanced-stage Hodgkin lymphoma: yes but which one?

**DOI:** 10.18632/oncotarget.27045

**Published:** 2019-07-09

**Authors:** Cédric Rossi, Olivier Casasnovas

**Affiliations:** ^1^ Department of Hematology, and INSERM UMR 1231, CHU F. Mitterrand, Dijon, France

**Keywords:** Hodgkin, advanced-stage, PET-CT, BEACOPP

In last decades, two regimen have been developed to treat young patients with advanced-stage Hodgkin lymphoma (HL) in the first-line setting: doxorubicin, bleomycin, vinblastine and dacarbazine (ABVD) and bleomycin, etoposide, doxorubicin, cyclophosphamide, vincristine, procarbazine, prednisone-escalated (BEACOPPesc). Overall, 70% and 85% of advanced-stage HL patients are cured with ABVD and BEACOPPesc respectively. However, acute and long-term adverse effects related to chemotherapy impact the patients outcome, significantly more frequently with BEACOPPesc than ABVD, then reducing toxicities has become one crucial criterion to chose and optimize the risk-benefit ratio of the treatment. Basically, 18-Fluoro-deoxyglucose positron emission tomography (PET) demonstrated a prominent place in the management of patients with HL for assessing both the baseline stage of the disease and the early chemosensitivity to the first-line treatment [1]. Indeed, several trials have attempted to improve the balance between risk and benefit of the treatment in patients with advanced HL by adjusting the number of cycles and intensity of chemotherapy according to early PET response.

Escalating strategies were designed to reinforce the treatment in patients with insufficient metabolic response after 2 cycles of ABVD and shift to BEACOPPesc. In the RATHL study [2] 3-year PFS and OS in the 16% patients with positive PET2, were 67.5% and 87.8% respectively, appearing better than patients pursuing ABVD in historical controls [3, 4], but lower than negative PET2 patients who achieved 86% 3-years PFS and 97% OS respectively. Similar results were observed in the SWOG S0816 [5] and GITIL trials [6], with consistent 64% 3-year PFS in the subset of roughly 18% positive PET2 patients, lower than the 82% PFS in negative PET2 patients [5]. The apparent better outcome of patients in the RATHL study was probably mainly due to a more favorable baseline profile as 41 % of the enrolled patients had an Ann Arbor stage II and 17% an IPS>3.

A second approach tested de-escalation therapy in metabolic responders after 2 cycles of BEACOPPesc (LYSA AHL2011 [7], and GHSG HD18 studies [8]). In AHL2011 study, 6 cycles of BEACOPPesc, were randomly compared to a PET-guided treatment delivering after 2 cycles of BEACOPPesc, 4 cycles of ABVD in negative PET2 patients and 4 additional cycles of BEACOPPesc in positive PET2 patients. PET2 positivity rate was less than 13%, and 84% of patients received the de-escalated therapy in the PET-guided arm which was associated with a significantly lower toxicity. Outcome of patients was similar in both arms and 3y-PFS was significantly lower in positive PET2 patients (70.7%) compared to PET2 negative patients (91.8%).

The HD18 [8] trial, randomly tested 4 cycles of BEACOPPesc for negative PET2 patients versus 6 to 8 cycles and addition of rituximab to BEACOPPesc for positive PET2 patients. Unfortunately, the inappropriate Deauville score cutoff (3 to 5) to declare PET positivity in this study leads to an excessive PET positivity rate of 48% and makes this study difficult to compare with other PET-guided treatments. However, rituximab did not bring any outcome improvement in positive PET2 patients. In the negative PET2 cohort, 3-year PFS was similar for 4 (95.3%) and 6/8 cycles of BEACOPPesc (91.7%), and a benefit in OS was observed (98.8% versus 95.7%, HR = 0.32, 95% CI 0.14-0.72) in patients receiving 4 cycles due to less toxic death. However, 4 cycles of BEACOPPesc seem still associated to more frequent grade 3 or higher hematological toxicity (anemia: 39% vs 24%, thrombocytopenia: 57% vs 36%) compared to 2 BEACOPPesc + 4 ABVD in AHL2011 study.

Then, should we start with ABVD or BEACOPPesc in PET-guided strategies and what could be the better option? A major issue is that no risk factor at baseline including IPS is able to guide the choice of upfront chemotherapy regimen as low risk patients benefit also from intensified upfront treatment [9]. Although cross-trial comparisons could be hazardous, PET-driven strategies after ABVD provide inferior results to those after BEACOPPesc developed in the AHL2011 study with less ability to control disease among a higher number of positive PET2 patients despite intensification of their treatment, and also a less good PFS in negative PET2 patients. The price to pay in terms of toxicity when applying the AHL2011 strategy is acceptable as most of the patients will receive deescalated treatment even if long term toxicity remains to be further analyzed.

Could new drugs such as brentuximab vedotin (Bv) and PD-1 blockers which demonstrated relevant clinical efficacy in relapsed HL, allow modifying the landscape of upfront treatment regimen in advanced HL? Bv and anti-PD1 were tested in combination with modified backbones of ABVD or BEACOPP chemotherapy regimen. However, while AVD-Bv [10] was shown to slightly improve the 2-year modified PFS compared to ABVD in a non PET-guided treatment (82.1% vs 77.2%) the survival achieved was disappointing and AVD-Bv was associated with more frequent toxicity including neuropathy and neutropenia. BrECADD, a combination of Bv introduced in a BEACOPPesc backbone, was shown to induce less toxicity than original BEACOPPesc and is currently randomly compared to BEACOPPesc in a PET-driven approach (GHSG HD21 study) (Clinical Trials.gov Identifier: NCT02661503). Nivolumab, a monoclonal antibody targeting PD-1, combined with AVD in a phase II trial which enrolled 51 newly diagnosed advanced HL patients [11] provided only 67% complete response after completion of 12 doses of N-AVD while 59% of patients experienced grade 3–4 adverse event predominantly neutropenia. The further outcome of these patients is pending as median follow-up was only 9.4 months.

Altogether, these recent results suggest that the best strategy to currently treat advanced Hodgkin lymphoma patients, allowing to optimize the disease control and minimize the therapy side effects is to offer a PET-guided approach after 2 cycles of BEACOPPesc shifting to ABVD for negative PET patients. Due to the excellent outcome of most patients with this approach, the aim to still improving these results with new alternative upfront treatments should be challenging and mainly focus on improving the treatment strategy of positive PET2 patients.
